# LDPE/Bismuth Oxide Nanocomposite: Preparation, Characterization and Application in X-ray Shielding

**DOI:** 10.3390/polym13183081

**Published:** 2021-09-13

**Authors:** Saad Alshahri, Mohammed Alsuhybani, Eid Alosime, Mansour Almurayshid, Alhanouf Alrwais, Salha Alotaibi

**Affiliations:** Nuclear Science Research Institute, King Abdulaziz City for Science and Technology, Riyadh 11442, Saudi Arabia; sshahri@kacst.edu.sa (S.A.); alosimi@kacst.edu.sa (E.A.); aalrwais@kacst.edu.sa (A.A.); sealotaibi@kacst.edu.sa (S.A.)

**Keywords:** polymeric nanocomposite, LDPE, Bi_2_O_3_, radiation shielding, attenuation of X-ray radiation

## Abstract

Recently developed polymer-based composites could prove useful in many applications such as in radiation shielding. In this work, the potential of a bismuth oxide (Bi_2_O_3_) nanofiller based on an LDPE polymer was developed as lead-free X-ray radiation shielding offering the benefits of lightness, low-cost and non-toxic compared to pure lead. Three different LDPE-based composites were prepared with varying weight percentages of Bi_2_O_3_: 5%, 10% and 15%. The characterizations were extended to include structural properties, physical features, mechanical and thermal properties, and radiation shielding efficiency for the prepared nanocomposites. The results revealed that the incorporation of the Bi_2_O_3_ nanofiller into an LDPE improved the density of the composites. There was also a slight increase in the tensile strength and tensile modulus. In addition, there was a clear improvement in the efficiency of the shield when fillers were added to the LDPE polymer. The LDPE + Bi_2_O_3_ (15%) composite needed the lowest thickness to attenuate 50% of the incident X-rays. The LDPE + Bi_2_O_3_ (15%) polymer can also block around 80% of X-rays at 47.9 keV. In real practice, a thicker shield of the proposed composite materials, or a higher percentage of the filler could be employed to safely ensure the radiation is blocked.

## 1. Introduction

The overall unique properties of various composite materials have recently attracted a wide range of scientific research regarding radiation protection due to the materials’ low weight, low manufacturing cost, mechanical strength, flexibility, and chemical stability [1]. Lead (Pb) has traditionally been employed as a shield since it has a high density and high efficiency in form of sheets, plates, foils, laminates, bricks, and blocks [2,3]. Nevertheless, the use of Pb is limited and it is unsuitable for some specific applications that require low cost, flexibility, chemical stability, mechanical strength, and lightness [4]. Polymer composites are an attractive option for radiation shielding because of their many advantages including being environmentally friendly, light, non-toxic, and flexible [5]. Consequently, diverse researchers have used various types of polymers as a matrix and have included fillers that provide reinforcement, depending on their overall application. The most used composites in X- and γ-rays shields are polymeric materials reinforced by metal and metal oxide [6,7,8,9]. As noted in the literature, metal–polymer composites generally combine two dissimilar components forming a light lattice together with Z particles [10]. The increased shielding properties of the metal–polymer are because of the uniform distribution of metal and metal oxide particles within the defined matrix [11].

Investigators have focused and reported several polymer matrices that can be used as X- and γ-ray shields. These polymer matrices include bismuth oxide (Bi_2_O_3_) filled poly (methyl methacrylate) composites, a high-density polyethylene (HDPE) composite that is loaded with tungsten (W), molybdenum sulfide (MoS_2_), boron carbide (B_4_C), micro-and nanosized tungsten oxide (WO_3_) dispersed emulsion polyvinyl chloride (EPVC) polymer composites, lead oxide filled isophthalic resin polymer composites, silicone rubber composites that contain bismuth content, polymer bricks, polyester composites that are reinforced with zinc, composites of HDPE with zinc oxide, lead oxide, and cadmium oxide [12,13,14,15,16,17,18,19,20,21]. The research attention has increasingly turned toward the overall effects of the presence of nanoparticles within the shielding materials, because of the novel uses of the materials [22]. The quantum effects and increase in the surface-to-volume ratio factor of nanoparticles were reported as the main factors that bring about dissimilar behaviors of the nano and microparticles. The outlined parameters affect the mechanical, thermal and shielding properties of the materials in some cases [23]. Because of induced agglomeration, the dispersion of nanoparticles into the overall polymer matrix is known to be more challenging than the dispersion of micro-sized particles [24]. Accordingly, the prominent features known to nano-scale particles than microparticles are known to cause an impressive increase in the resultant attenuation coefficient. As asserted in various studies, combining two phases of matrix and reinforcement is more complex and diverse than each individual phase [25].

Many researchers have performed studies on Pb-free composite shields. For example, one study designed and fabricated composites that consisted of HDPE mixed with micro-sized and nano-sized cadmium oxide particles for attenuation of photon beams with energy that ranged from 59.53 keV up to 1408.01 keV [19]. It was established that the nanoscale reinforced HDPE enhanced the overall shielding properties, particularly at lower photon energies. Another study was conducted on a new polyvinyl alcohol (PVA)/WO_3_ composite based on the Monte Carlo N-Particle (MCNP) simulation code [26]. It was noted that the PVA/WO_3_ composite could be considered as a shield for photon energy at the levels of 662, 778, 964, 1112, 1170, 1130, and 1407 keV. In addition, Atashi et al. conducted some fabrication of flexible silicone rubber/W/Bi_2_O_3_ using the technique of open mold cast [5]. The final composites possessed higher attenuation coefficients. Furthermore, it was established that increasing Bi_2_O_3_ in composites decreases the agglomeration of fillers. However, the composite of PVA containing 0–40 wt% filler loading (Bi_2_O_3_ and WO_3_) used in X-ray shielding is reported to play an important role in determining the density and the thickness of the composite sample [9].

Varying the quantity of tungsten nanopowder yielded improvement in thermophysical, radiation shielding and mechanical properties by [27]. In addition, concentration ranges of 30–70 wt% Bi_2_O_3_ were dispersed in carboxylated nitrile butadiene rubber latex films [28]. The films effectively attenuated low-energy photon beams. The overall effect of density on the composites’ suitability as radiation shield was studied using X-rays [29]. The attenuation performance against radiation was studied by varying the amounts of powdered fillers lead oxide and WO_3_ added to low-density polyethylene (LDPE). The results of the study indicated that samples with higher filler loads showed good attenuation performance against radiation [30].

In the current study, LDPE resin was chosen as a matrix duet to its superior mechanical and chemical properties. Bi_2_O_3_ was chosen for an alternative X-ray protective filler since it has key potential properties such as high density, high melting point, low conductivity, and is available in fine powder form. Therefore, the primary experiments aimed at furthering the understanding of LDPE nanocomposites. LDPE + Bi_2_O_3_ composites with different loadings of nano Bi_2_O_3_ were prepared via melt compounding. The study then conducted a study of X-ray attenuation, structure, mechanical, and thermal characteristics of Bi_2_O_3_ filled polymer composites. The results from this work would not only offer exciting possibilities for new, effective and safe X-ray protective LDPE nanocomposites but also highlight the potential of these new composite materials to be further developed for radiation shielding applications.

## 2. Materials and Methods

### 2.1. Materials

A commercial sample of LDPE (density: 0.93 g cm^−3^) was obtained from the Saudi Arabian Basic Industries Corporation (SABIC),(Riyadh, Saudi Arabia), under the trade name LDPE HP 0722N. Bismuth oxide nano powder was obtained from (Alfa Aesar, Kandel, Germany) with particle size smaller than 100 nm.

### 2.2. Nanocomposite Preparation

The Xplore conical twin-screw extruder was used to prepare the following samples: Pure LDPE, 5 wt%, 10 wt% and 15 wt% of Bi_2_O_3_ for LDPE + Bi_2_O_3_ nanocomposites. Firstly, an electrical balance (Sartorius Analytical, Karlsruhe, Germany) weighted LDPE and Bi_2_O_3_ with an accuracy ± 0.0001 g. Subsequently, 20 g of LDPE with a specified weight percentage of Bi_2_O_3_ was fed into a mini twin-screw extruder at 170 °C, for 10 min. The rotator speed was set to 100 rounds per minute (rpm) to ensure a uniformly mixed composite.

The fully mixed sample was then put into a stainless-steel frame (100 × 100 × 1 mm^3^) to be hot presses between two layers. The pressing was carried out by using a hydraulic press preheated at 170 °C for 3 min. The pressure was then gradually raised to 100 kN for another 10 min. The sample was left in the press for 20 min to cool down to room temperature. Finally, the resulting sheet was taken out of the mold and cut into circular samples of 25 mm in diameter to perform radiation-shielding tests and into dumbbell shape for tensile testing according to ASTM D638.

## 3. Characterization

### 3.1. X-ray Differentiation

X-ray diffraction (XRD) analysis was carried out using JOEL instruments (Tokyo, Japan) using Cu Kα radiation (λ = 0.1540 nm, a tube operated at 40 kV and a filament current of 40 mA, the Bragg’s angle (2θ) was in the range of 5°–80° using 0.01 step with and 1 s time count). All data were recorded and analyzed using the machine software. The crystal size was calculated by the Scherer equation:

(1) $$D=\frac{K\;\lambda }{\beta \;\cos\;\theta }$$

where D is the crystallite size (nm), K is the crystallite shape factor (K = 0.9), λ is the X-ray wavelength of Cu (equal =0.154 nm) and β is the full width half maximum (FWHM) of XRD diffraction peak in radians.

### 3.2. Scanning Electron Microscope (SEM)

The surface morphology and dispersion of the nanocomposite were studied by SEM using JEOL (Tokyo, Japan). The samples were prepared as follows: the specimen was stuck on a 10 mm diameter carbon tab attached to the top of the aluminum pin tube. Then, the sample was coated with gold using a sputter coater. The coating exposure was 2 min.

### 3.3. Density Measurements

The experimental and calculated densities were evaluated using the Archimedes method (water as an immersing medium) for the mixture. The relative density of pure Bi_2_O_3_ and LDPE were calculated to 8.9 and 0.93 g.cm^−3^ respectively using the Mettler Toledo XS204 instrument (Greifensee, Switzerland).

### 3.4. Tensile Testing

Tensile testing was conducted by a tensile machine (Instron 5982, Grove city, PA, USA) according to ASTM D638, (dumbbell samples cut from pressed sheets 1 mm thickness) at crosshead speed 50 mm min^−1^. The results are the average of at least five measurements. The tensile strength, Young’s modulus and elongation at the break of the composite were calculated.

### 3.5. Thermal Analysis by TGA

The thermal behavior of the sample was evaluated using a thermogravimetric analyzer (TGA 1, Perkin Elmer, Shelton, CT, USA). Each sample was heated from room temperature to 700 °C at a rate of 10 °C min^−1^.

## 4. Results and Discussion

### 4.1. Density Measurements

In the present study, density was used to represent the physical property of LDPE composites. A high-density metal oxide nanofiller was used to improve some properties of the respective thermoplastic composition and density. Table 1 illustrates the test results via the resultant density of LDPE composites taken as a function of Bi_2_O_3_ content. Considering the rule of mixture, the overall density of any given particulate-filled composite is known to be related to the density of its constituent particles. As noted in Table 1, the overall density of the composite increased as the amount loading of Bi_2_O_3_ increased. The increase can be linked to the density range of Bi_2_O_3_ which is 8.9 g cm^−3^: much higher than LDPE’s density of 0.93 g cm^−3^. The density range of LDPE composites was between 0.961–1.060 g cm^−3^. Ambika et al. in which the density of unsaturated polyester (UP) was found to increase the overall increase in the Bi_2_O_3_ filler content reported a similar phenomenon [31]. This was related to the fact that Bi_2_O_3_ has a higher density compared to UP. As explained by Pavlenko et al., increasing the Bi_2_O_3_ nanoparticle filler content within the polyimide (PI) composites results in an increase in the density of PI, since Bi_2_O_3_ is known to have a higher density compared to other formulation contents [32]. In the experiment, the highest reported density was 1.07 g cm^−3^ for an LDPE with 15 wt% of Bi_2_O_3_, yielding an overall increment of approximately 13% in density in comparison to pure LDPE. On the other hand, composites of an LDPE with 5 wt% Bi_2_O_3_content showed the lowest density, at about 0.961 g cm^−3^.

### 4.2. Thermal Stability

**Figure 1:**
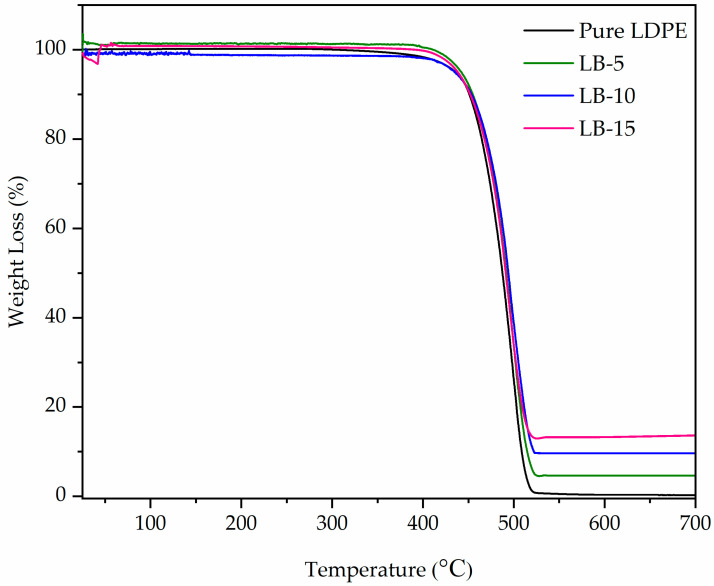
TGA thermograms for pure LDPE and LDPE/ Bi_2_O_3_ composites.

In this study, an overall determination of thermal characteristics was carried out between 25–700 °C. Figure 1 illustrates the TGA curves of pure LPDE and those of the composites containing up to 15 wt% of Bi_2_O_3_. Figure 1 affirms that pure LDPE is stable up to 459.1 °C without the general loss of mass on the respective TGA curve. Complete thermal decomposition of the LDPE occurs at 500 °C. The introduction of inorganic filler results in a significant increase in the overall thermal stability of the respective polymers [33,34,35]. This is exemplified clearly by LDPE + Bi_2_O_3_ (15%). In the end, an increase in the Bi_2_O_3_ content results in a decrease in the rate of mass loss for the composites. Nonetheless, a slight mass loss in the composites starts at 80–120 °C. This is related to the loss of sorbed-water and hydroxyl water that is contained in the Bi_2_O_3_ [36]. The data on the thermal stability of various composites are outlined in Table 2. It can be concluded from the data in Table 2 and Figure 1 that the overall thermal stability of the LDPE is generally less than that of LDPE + Bi_2_O_3_ nanocomposites. This is a clear confirmation of the fact that the thermal stability of various inorganic compounds is greater than those of polymers. Furthermore, the overall increase in thermal stability of such composites could be linked to an increase in the density of Bi_2_O_3__._ In the experiment, the char yield of LDPE + Bi_2_O_3_ nanocomposite was found to be 4.6%, 9.6%, and 13.2% (respectively) higher than that of respective pure LDPE that was 0.3% at 600 °C. The reported values affirm that the dispersion of Bi_2_O_3_ into the polymer was indeed great thus closer to the initial percentage of weight added. Table 3 outlines the burning characteristic of LDPE that contained various loadings of Bi_2_O_3_. The present study assessed whether the overall presence of metal oxide in relation to the composition was consistent with the outlined theoretical quantity of Bi_2_O_3_. Considering the range of experimental error, the estimated amount of metal oxide is in agreement with the loading weight percentage of Bi_2_O_3_ to LDPE.

### 4.3. Mechanical Properties

**Figure 2:**
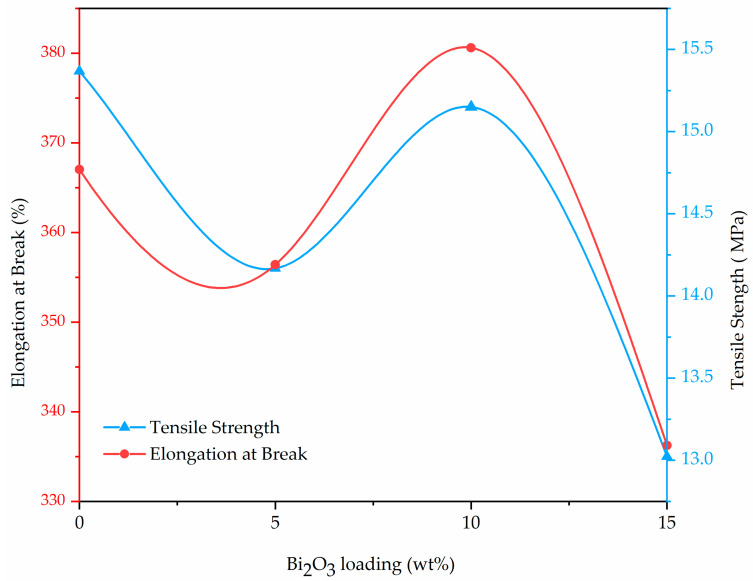
Effect of wt% of Bi_2_O_3_ on the mechanical properties of LDPE.

Table 4 illustrates mechanical properties such as tensile strength, elongation at break and Young’s modulus of LDPE composites taken as a function of Bi_2_O_3_ content, as shown in Figure 2. From Table 4, it can be inferred that all mechanical properties of various composites decreased with the overall increase in the Bi_2_O_3_ filler content excluding LDPE + Bi_2_O_3_ (10%). This could be attributed to the fact that the low tensile strength of the LPDE compound being used in the experiment. The highest tensile strength of 15.51 MPa was achieved with the LDPE composite that had 10 wt% Bi_2_O_3_ nanofiller. However, an increase in Bi_2_O_3_ filler loading over 10 wt% reduced the tensile strength. This could be due to the poor adhesion that exists between filler particles and the requisite LDPE matrix which weakened the resultant interfacial zone between the polymer and the particles of Bi_2_O_3_. The outlined weak zone increased with an increase in filler content, thus decreasing the tensile strength and elongation at the break of the composite [37]. Young’s modulus was computed from the slope at zero percent within the tensile curve. The outlined trend was similarly observed within the LDPE + Bi_2_O_3_ (10%): there was a slight increase in Young’s modulus with the adding 10 wt% filler content. In essence, the incorporation of Bi_2_O_3_ nanoparticles can enhance the stiffness of LDPE but an additional increase in particle loadings did not lead to a substantial enhancement to the resultant Young’s modulus. Ideally, agglomerations take place at higher particle loading thus reducing the total surface area of the respective nanoparticles [38].

### 4.4. X-ray Diffraction

**Figure 3:**
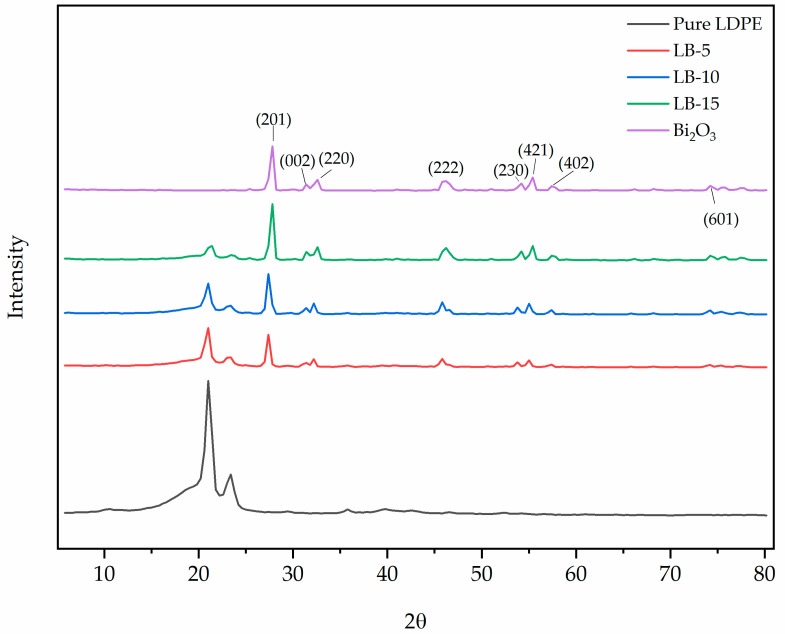
XRD patterns of pure LDPE, Bi_2_O_3_ and its composites.

The XRD patterns for pure Bi_2_O_3_, pure LDPE, and several quantities of Bi_2_O_3_ with LDPE are displayed in Figure 3. The appearance of diffraction peaks/shoulders at 2θ = 27.80°, 31.73°, 32.70°, 47.20◦, 55.22°, 56.48°, 57.74°, and 76.47° is consistent with the dominant diffraction 2θ angles of the β-phase of tetragonal Bi_2_O_3_ crystal structure [4]. Figure 3 outlines numerous peaks that correspond to the orthorhombic unit cell of polyethylene. For these peaks, the lattice parameters include a=7.39 Å, b=4.93 Å, and c=2.52 Å. The unit cell structures of 110, and the reflection of 200 planes, have two main peaks at 21° and 24° respectively. The results that were obtained in this experiment confirm the results of the previous study [4]. On the other hand, two other peaks are known to be located at 30° and 36°, which correspond to reflection planes of 210 and 020, respectively [39,40]. What is more, other weak peaks are located ranging from 40° to 60° [40]. As such, the role of Bi_2_O_3_ with LDPE can be assumed affirmatively. In essence, the addition of Bi_2_O_3_seems under slight variation in its position relative to the main peaks at 21° and 24°. Even so, the accumulation of Bi_2_O_3_ affected the overall original LDPE structure significantly. There is a new peak at approximately 28° that is characteristic of a high quantity of bismuth concentration and is linked to the reflection plane 120. Table 5 outlines the values of crystallite size computed using Scherrer’s equation. From the calculations, the crystalize size of Bi_2_O_3_ within the LDPE matrix as computed by Scherrer’s equation ranges from 18.39 nm to 18.49 nm. A closer look at the results reveals no considerable difference in the respective crystallite size of the filler particles. The noted slight difference in the overall crystallite size can be attributed to the crystallite size effect.

### 4.5. Morphology

**Figure 4:**
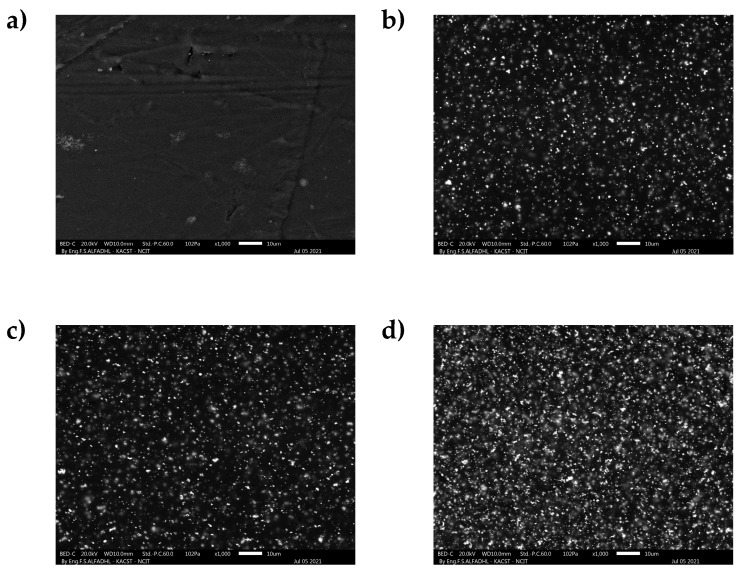
The surface morphology of LDPE nanocomposites (**a**) pure LDPE (**b**) 5 wt% Bi_2_O_3_ (**c**) 10 wt% Bi_2_O_3_ and (**d**) 15 wt% Bi_2_O_3_.

The surface morphology was determined by SEM. The morphology of various LDPE composites that have 0 wt%, 5 wt%, 10 wt% and 15 wt% Bi_2_O_3_ addition is shown in Figure 4 (Details regarding the density of Bi atoms in the nanocomposites according to the analysis of SEM-EDS images are shown in the Supplementary Materials, Figures S1–S4). There are no observable particles on the surface of the pure LDPE composite; nonetheless, in the experiment, there was an observed even distribution of particles that dispersed more densely with increased loading, especially in the case of LDPE composites that had 5 wt%, 10 wt% and 15 wt% added Bi_2_O_3_. The dispersion quality of the nanoparticles into the LDPE was observed to differ based on the overall concentration of the nanoparticle. It is possible that some particles could have settled down as the mixture settled because of the difference in chemical structure and physical properties of both the polymer and nanoparticles despite the mixing process being uniform. This could further be attributed to higher density and/or lack of interaction or bonding with various polymer pellets during the heating process [41]. Nevertheless, the composite that had a 15 wt% filler load showed the agglomeration of filler particles thus forming larger particles. This must be minimized for the composites to perform better.

## 5. Shielding Properties and Application

### 5.1. Shielding Efficiency of the Polymer Composites in Low Energies Applications

**Figure 5:**
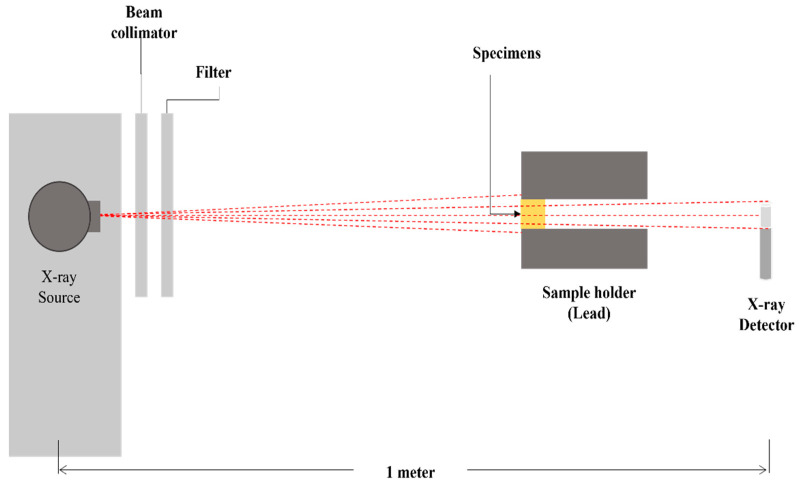
The experimental setup used for shielding efficiency proposed composite materials.

In Equation (2), the Lambert–Beer law is used to determine the attenuation characteristics of a shielding material by calculating the linear attenuation coefficient (µ) (cm^−1^) or mass attenuation coefficient (*µ*_m_ or *μ*/*ρ*) (cm^2^ g^−1^)

(2) $$\frac{I}{I_{o}}=e^{-\left(\frac{\mu }{\rho \;}\right)\rho x}$$

where *I_0_* is the initial intensity and *I* is the remaining radiation intensity (*I*) after traversing a layer (*x*) of the composite material considering the density of the absorber (ρ). The experiment evaluated *µ* and *µ*_m_ of the Bi_2_O_3_ composite samples based on the LDPE polymer and the impact of the concentration of the Bi_2_O_3_ materials on the polymers. We used an X-ray source and an ionization chamber detector configuration (Figure 5) in a sequence of irradiation. The discs sample holder was placed in between to estimate the intensity of the attenuated beams. Three filler concentrations of LDPE (5%, 10%, and 15%) were prepared in discs with a diameter of 2 cm each. The irradiation procedures were conducted using 1.80 cm^2^ collimator size and 30 s acquisition time. The µ values for the composite samples were determined using the intensity values with no disks and with discs for *I_o_* and *I* determination, respectively.

**Figure 6:**
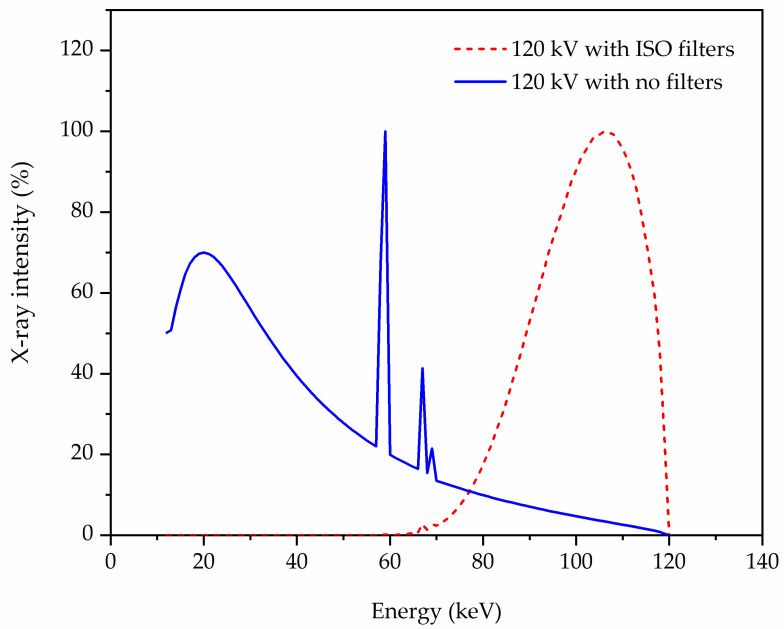
The distribution of the number of X-rays for 120 kVp extracted from SpekCalc with no filters and with ISO filter.

Table 6 shows the X-ray irradiation qualities used using a narrow beam spectrum condition allowing only primary photons to pass through the attenuating material to contribute to the detected signal [42]. Using SpekCalc software and for illustration, Figure 6 confirms the effective energy stated in ISO-4037 for the applied voltage of 120 kVp with filters provided in Table 6.

To validate our outcomes, the experimental values of the *µ*_m_ were obtained for the studied samples against energy (E) and were then compared to the calculated values (XCOM database) [43,44].

The attention efficiency of studied samples could be further examined by the half-value layer (HVL) and mean free path (MPF). The definition of these radiation protection quantities could be the thickness of the examined material needed to reduce the primary radiation to 50% and 36.8% respectively. They depend highly on energy and it can be said that efficient shielding has low HVL and MFP values.

(3) $$HVL=\frac{0.693}{\mu }$$

(4) $$MFP=\frac{1}{\mu }$$

The radiation protection efficiency (RPE) could be also used as an indication of the shielding ability of the composite samples by knowing the intensity values measured with and without samples [14]:

(5) $$\mathrm{RPE}=\left(1-\frac{I}{I_{o}}\right)\times 100\;\;\;\left(\%\right)$$

### 5.2. Shielding Ability Investigation of the Polymer Composites

**Figure 7:**
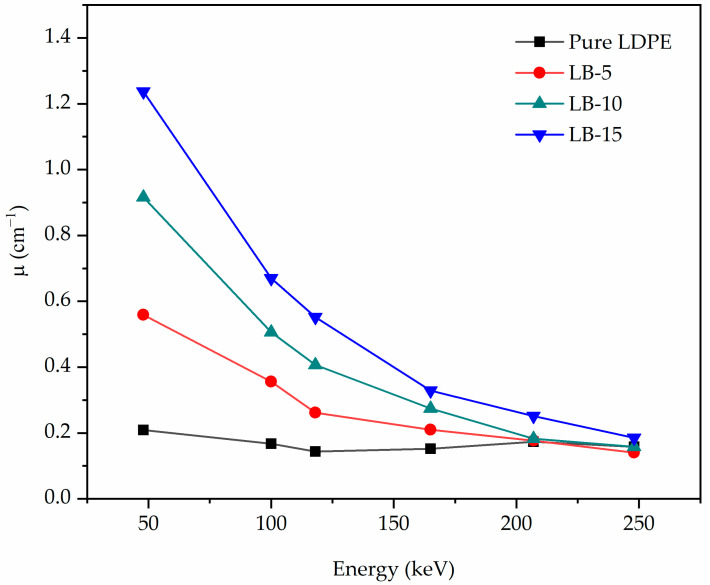
The measured linear attenuation coefficient against incident beam energy.

The shielding ability of composites based on the LDPE polymer was examined in attenuating 40.9–248 keV X-ray beams. The composite materials were developed, mixed with different percentages of Bi_2_O_3_ as filler, and eventually shaped into discs. The setup in Figure 5 used experimentally using narrow-spectrum X-ray beams to determine *I_o_* and *I* when the samples were placed, allowing to calculate the corresponding µ at different incident beam energies using Equation (2). Figure 7 shows the behavior of the measured *µ* against energy for the studied composites based on LDPE polymer prepared in different filler percentages of 5%, 10% and 15%. The µ values were observed to decrease with energy and the composite coded LB-15 containing 15% of the Bi_2_O_3_ possesses the highest attenuation across all energies of the incident beams compared to other composites. It is clear that the fillers have enhanced the efficiency compared to pure LDPE especially in low energy below 200 keV region where the predominant mechanism is photoelectric absorption which has a strong dependency on the incoming photon energy (1/E^3^) and the atomic number of the material (Z^4.5^) [45].

For verification purposes of the experimentally measured results, Table 7 illustrates the measured values of *µ*_m_ and the calculated results of *µ*_m_ extracted from the XCOM calculator based on the NIST database for the present composite samples. It was revealed that the measured results of µ_m_ and the calculated *µ*_m_ were close with an average error of 7.4%. The disagreements could result from experimental errors such as disc placement, the composites densities and the dispersion quality of the fillers in the matrix affecting the evaluation of *µ* values and eventually *µ*_m_.

**Figure 8:**
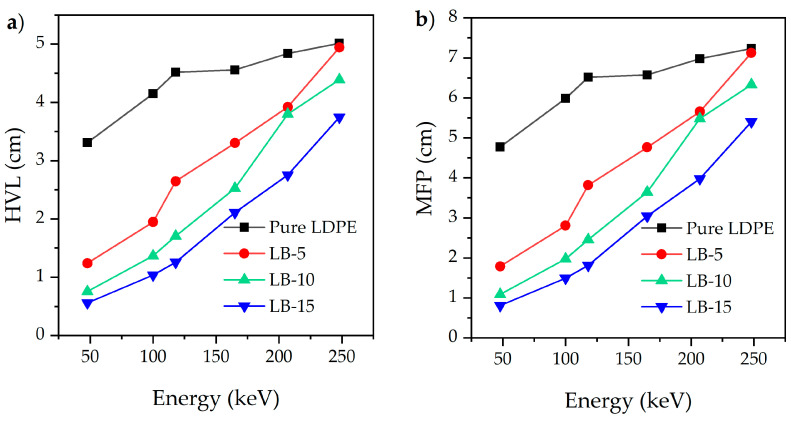
(**a**) HVL and (**b**) MFP values against energy for the composite samples prepared in this study.

The attenuation ability of a shield could be illustrated using the HVL and MFP values in Figure 8a and Figure 8b. In this examination, the outcomes show that a thinner thickness of fillers is needed to attenuate the X-ray beams compared to the pure LDPE polymer. Particularly, the thickness of pure LDPE required to absorb 50% of the X-rays is almost four times the thickness of the LDPE + Bi_2_O_3_ (15%) composite.

The results of RPE in Table 8 show that around a 1.60 cm thick shield of the composite with 15% filler could reduce approximately 40% of the incident beam at 100 keV. Increasing the thickness of the samples would allow them to be a potential choice for X-ray shielding in diagnostic radiology departments where the energy is used commonly below 100 keV at hospitals using kilovoltage X-rays.

The material will have better attenuation ability if it has lower HVL and MFP values. Figure 8 clearly shows that the HVL and MFP are energy-dependent. Therefore, the HVL and MFP values of the composite sample with 15 wt% of Bi_2_O_3_ with the best performance against radiation were chosen for comparison purposes with other materials or composites in the literature at 100 keV. The comparison results in Table 9 showed the Bi_2_O_3_-LDPE composites possess low values of HVL and MFP meaning better attenuation ability among other materials.

## 6. Conclusions

This study aimed to characterize a potential Pb-free and light-weighted shielding material based on LDPE polymer. The different weight percentages of the components of Bi_2_O_3_ within the final products could be linked to the variation in densities of the composites. The LDPE + Bi_2_O_3_ (15%) that had 15 wt% had higher bismuth composition compared to its LDPE counterparts. This can be affirmed by the SEM images which showed that the interaction between the polymer and the respective bismuth oxide can result in homogenous distribution and dispersion. Analysis using XRD revealed that the crystallite size ranged from 18.39 nm to 18.49 nm as determined based on Scherrer’s equation. The resultant minor difference could be attributed to the crystallite size effect. In the TG curves of the entire composites, one-stage degradation is present. The first degradation temperature is 459 °C that shows thermal stability until 600 °C.

The attenuation efficiency of the proposed materials was tested in terms of *µ*, *µ*_m_, HVL, and MFP by measuring the transmitted radiation through the proposed shields using a photon energy range up to 248 keV. Clear enhancement of the attenuation ability was observed when the percentage of filler to the polymer was increased specifically below 150 keV. A future direction of this study could be utilizing the proposed materials to determine their performance in higher energy ranges and neutrons. In addition, increasing the thickness of the proposed composite samples or increasing the percentage of the filler in the polymer could offer a potential shield for low-energy radiation application.

## Figures and Tables

**Table 1:** The density results of LDPE and its composites.

Sample Code	Composition (wt%)	Density (g cm^−3^) (Experimental)	Density (g cm^−3^) (Calculated)	Error (%)
Pure LDPE	Pure LDPE	0.926	0.930	0.430
LB-5	LDPE (95%) + Bi_2_O_3_ (5%)	0.961	0.973	1.293
LB-10	LDPE (90%) + Bi_2_O_3_ (10%)	1.010	1.021	3.270
LB-15	LDPE (85%) + Bi_2_O_3_ (15%)	1.060	1.074	8.762

**Table 2:** Summary of the TGA thermograms for pure LDPE and LDPE + Bi_2_O_3_ composites.

Sample	Bi_2_O_3_ Loading (wt%)	Onset Temp (°C)	High Peak Temp (°C)	Weight Loss at 600 °C (%)
Pure LDPE	0	459	500	0.3
LB-5	5	459	500.2	4.6
LB-10	10	461	501.2	9.6
LB-15	15	454	500.6	13.2

**Table 3:** Burn test of pure LDPE and LDPE + Bi_2_O_3_ composites.

Sample	Bi_2_O_3_ Loading (wt%)		Error (%)
	The Theoretical Values	The Experimental Values	
Pure LDPE	0	0	0
LB-5	5.00	4.49	10.2
LB-10	10.0	9.16	8.37
LB-15	15.0	14.5	3.39

**Table 4:** Results of mechanical properties of LDPE and its composites.

Sample	Bi_2_O_3_ Loading (wt%)	Tensile Strength (MPa)	Elongation @ Break (%)	Young’s Modulus (MPa)
Pure LDPE	0	14.77 ± 0.60	378 ± 19	355 ± 36
LB-5	5	14.19 ± 1.29	356 ± 18	355 ± 13
LB-10	10	15.51 ± 0.43	374 ± 22	378 ± 16
LB-15	15	13.09 ± 0.98	335 ± 48	350 ± 14

**Table 5:** Crystallite size of the LDPE composites.

Sample	2θ	Scherrer Crystallite Size (nm)	FWHM
Pure LDPE	21	9.52	0.8400
Pure Bi_2_O_3_	27.8	18.41	0.4400
LB-5	27.4	18.39	0.4400
LB-10	27.4	18.39	0.4400
LB-15	27.8	18.41	0.4400

**Table 6:** Narrow X-ray spectrum qualities used (Adopted from ISO-4037 [42]).

Shortened Name	Tube Potential (kV)	Effective Energy (keV)	Additional Filtration Thickness			
			mm Pb	mm Sn	mm Cu	mm Al
N-60	60	47.9	-	-	0.631	3.912
N-100	100	83.3	-	-	5.027	3.920
N-120	120	100	-	1.013	5.027	3.950
N-150	150	118	-	2.605	-	3.903
N-200	200	165	1.028	3.004	2.032	3.901
N-250	250	207	3.099	2.062	-	3.925
N-300	300	248	5.152	3.016	-	3.929

**Table 7:** The experimental and calculated values of the mass attenuation coefficient.

Energy (keV)	Pure LDPE		LB-5		LB-10		LB-15	
	Exp.	Cal.	Exp.	Cal.	Exp.	Cal.	Exp.	Cal.
47.90	0.2094 ± 0.0035	0.1987	0.5589 ± 0.0041	0.5775	0.9164 ± 0.0062	0.9562	1.2369 ± 0.0077	1.3350
100.00	0.1671 ± 0.0024	0.1687	0.3560 ± 0.0030	0.4086	0.5061 ± 0.0030	0.6485	0.6696 ± 0.0032	0.8883
118.00	0.1535 ± 0.0022	0.1620	0.2619 ± 0.0027	0.3171	0.4067 ± 0.0028	0.4721	0.5520 ± 0.0029	0.6271
165.00	0.1520 ± 0.0017	0.1478	0.2010 ± 0.0019	0.2109	0.2745 ± 0.0025	0.2739	0.3287 ± 0.0024	0.3369
207.00	0.1432 ± 0.0011	0.1378	0.1768 ± 0.0017	0.1716	0.1824 ± 0.0025	0.2053	0.2515 ± 0.0023	0.2391
248.00	0.1382 ± 0.0020	0.1298	0.1403 ± 0.0014	0.1501	0.1579 ± 0.0022	0.1704	0.1849 ± 0.0020	0.1908

**Table 8:** The RPE values in % for the samples prepared in this study.

Energy (keV)	Pure LDPE	LB-5	LB-10	LB-15
47.90	17.11	44.17	76.52	79.16
100.00	14.02	23.17	46.95	40.24
118.00	13.90	31.02	55.08	57.22
165.00	12.06	23.90	47.43	50.34
207.00	12.73	19.66	35.21	34.08
248.00	14.37	16.84	25.05	27.31

**Table 9:** Comparison of the measured HVL and MFP outcomes with other materials in the literature at 100 keV.

Study	Composites	HVL (cm)	MFP (cm)
This study	LDPE 85% + Bi_2_O_3_ 15%	1.03	1.50
Almurayshid, et al. 2021 [45]	HDPE 85% + W 15%	1.18	1.63
Almurayshid, et al. 2021 [45]	HDPE 85% + Mo 15%	2.29	3.30
(Alavian and Tavakoli-Anbaran, 2019) [46]	LDPE 99% + W 1%	-	4.27
(Gurler and Akar-Tarim, 2016) [47]	Concrete (NBS)	1.81	2.62

## Supplementary Materials

The following are available online at https://www.mdpi.com/article/10.3390/polym13183081/s1, Figure S1: SEM-EDS images of pure LDPE, Figure S2: SEM-EDS images of LDPE composite containing a 5 wt% loading of Bi_2_O_3_, Figure S3: SEM-EDS images of LDPE composite containing a 10 wt% loading of Bi_2_O_3_, Figure S4: SEM-EDS images of LDPE composite containing a 10 wt% loading of Bi_2_O_3_.
