# Supplementary material for: LDPE/Bismuth Oxide Nanocomposite: Preparation, Characterization and Application in X-ray Shielding
Source: Polymers (Basel). 2021 Sep 13;13(18):3081. doi: 10.3390/polym13183081 (PMC8471621; doi:10.3390/polym13183081)
Supplement: Supplementary file 1 [file polymers-13-03081-s001.zip › polymers-1373212-supplementary.pdf]

LDPE/Bismuth Oxide Nanocomposite: Preparation, Characterization and Application in X-ray Shielding

Saad Alshehri, Mohammed Alsuhybani *, Eid Alosime, Mansour Almurayshid *, Alhanouf Alrwais and Salha Alotaibi

Nuclear Science Research Institute, King Abdulaziz City for Science and Technology, Riyadh 11442. Saudi Arabia; sshahri@kacst.edu.sa(S.A.); alosimi@kacst.edu.sa(E.A.); aalrwais@kacst.edu.sa(A.A.); sealotaibi@kacst.edu.sa(S.A.)

* Corresponding Authors: sohybani@kacst.edu.sa(M.A.), malmurayshid@kacst.edu.sa(M.A.)

SEM – EDS analysis has been performed to provide further evidence for the number and density of Bi atoms in the LDPE nanocomposites.

Figures S1-S4 show the results of SEM-EDS analysis, which have performed for LDPE containing different weight percent of Bi_2O_3: 0 wt%, 5 wt% , 10wt% and 15 wt%.

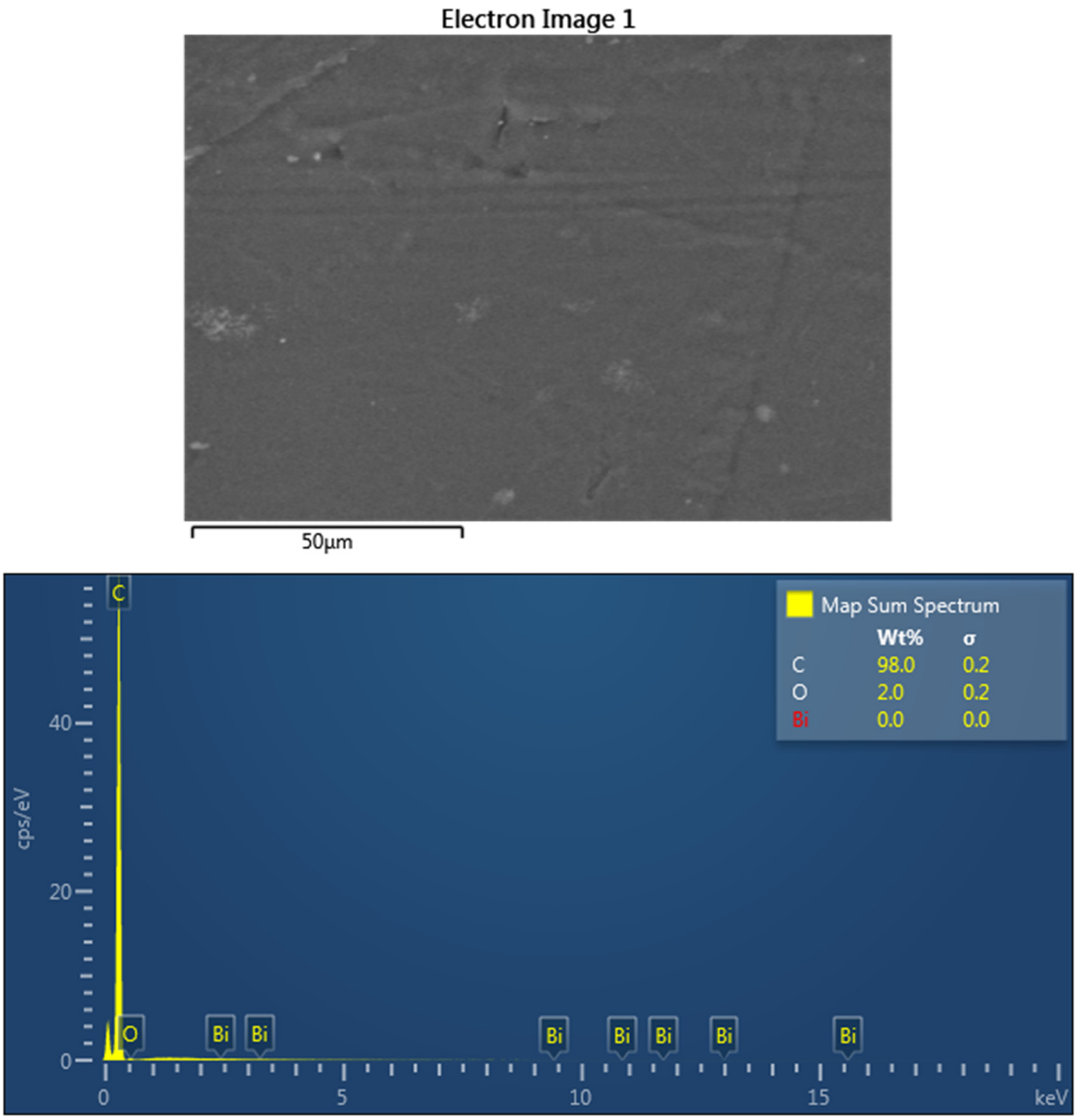

Element	Wt%	Wt% Sigma
C	98.02	0.16
O	1.98	0.16
Bi	0.00	0.00
Total:	100.00	

Figure S1. SEM-EDS images of pure LDPE.

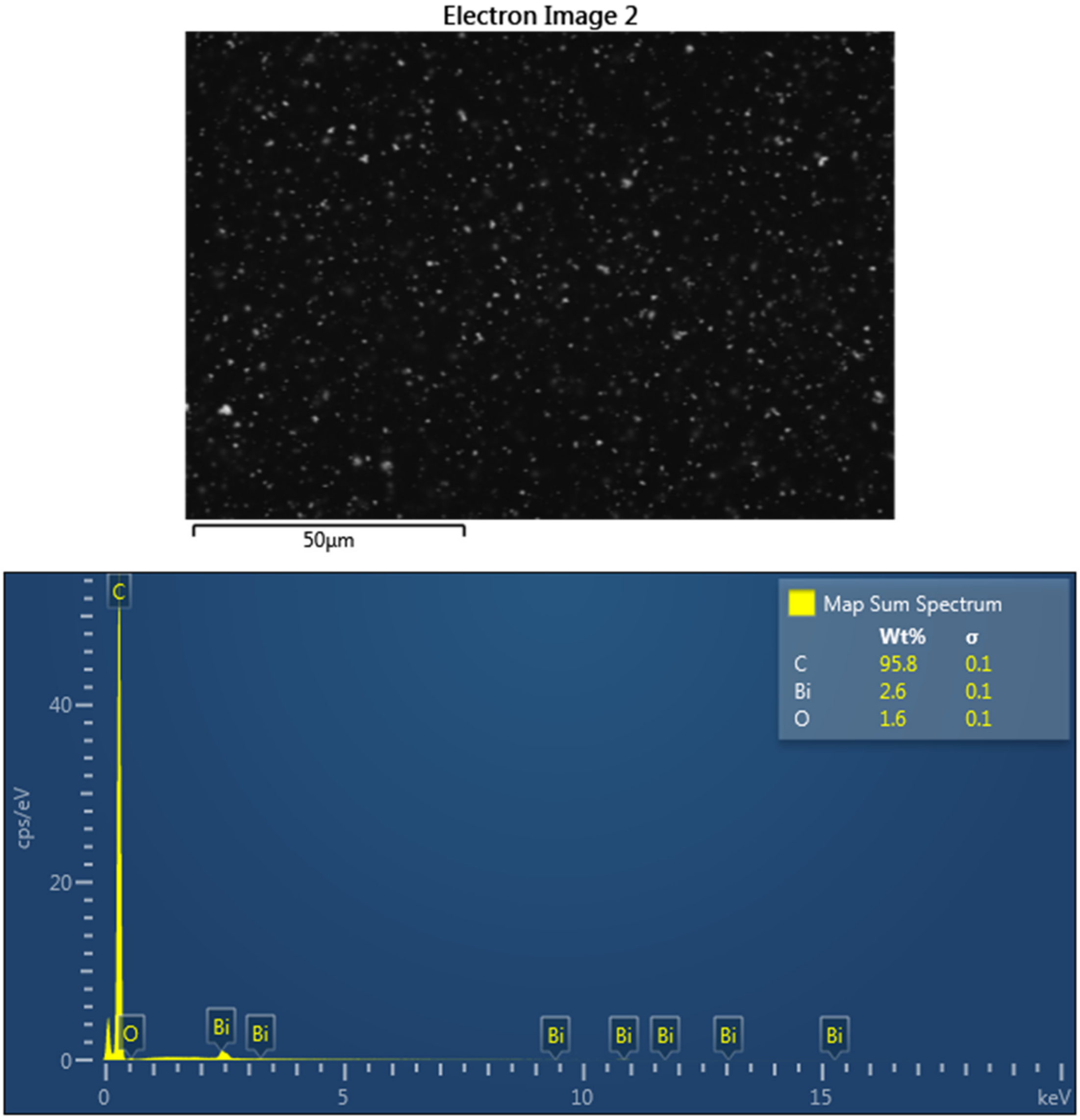

Element	Wt%	Wt% Sigma
C	95.77	0.11
O	1.64	0.09
Bi	2.59	0.07
Total:	100.00	

Figure S2. SEM-EDS images of LDPE composite containing a 5 wt% loading of Bi_2O_3.

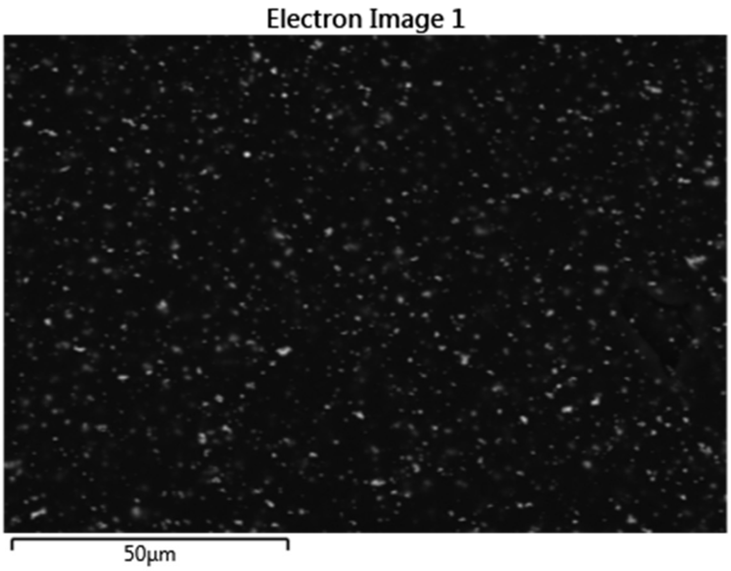

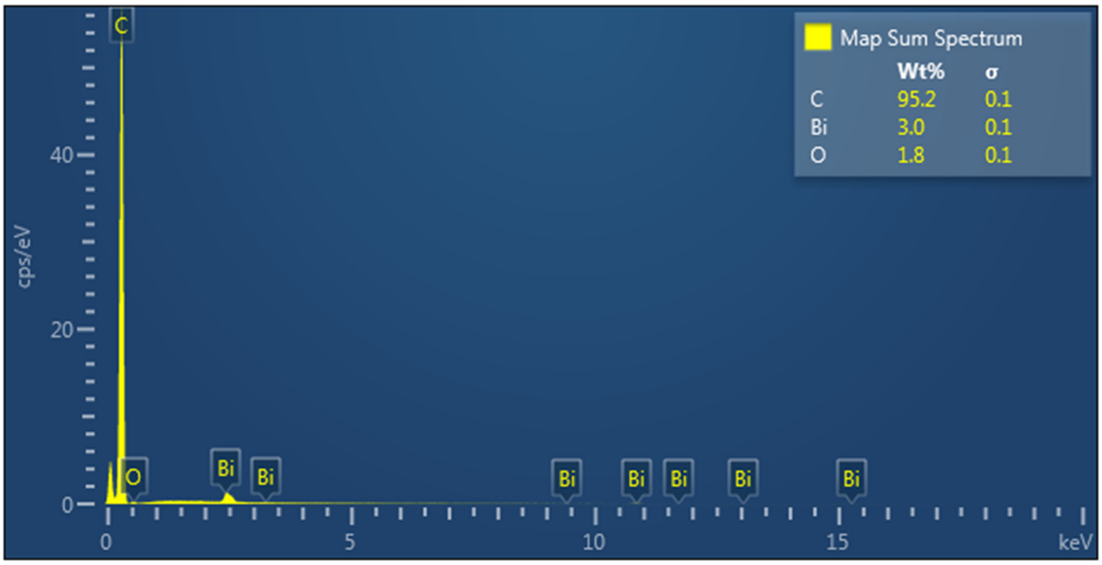

Element	Wt%	Wt% Sigma
C	95.19	0.13
O	1.77	0.10
Bi	3.04	0.08
Total:	100.00	

Figure S3. SEM-EDS images of LDPE composite containing a 10 wt% loading of Bi_2O_3.

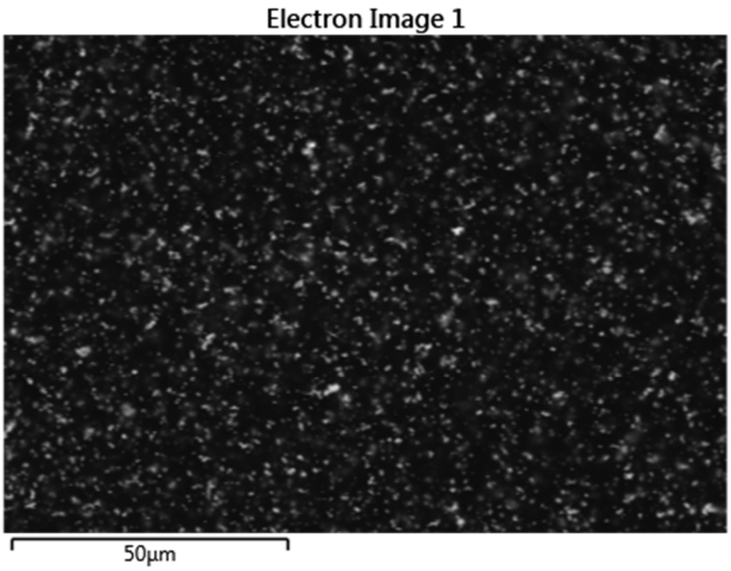

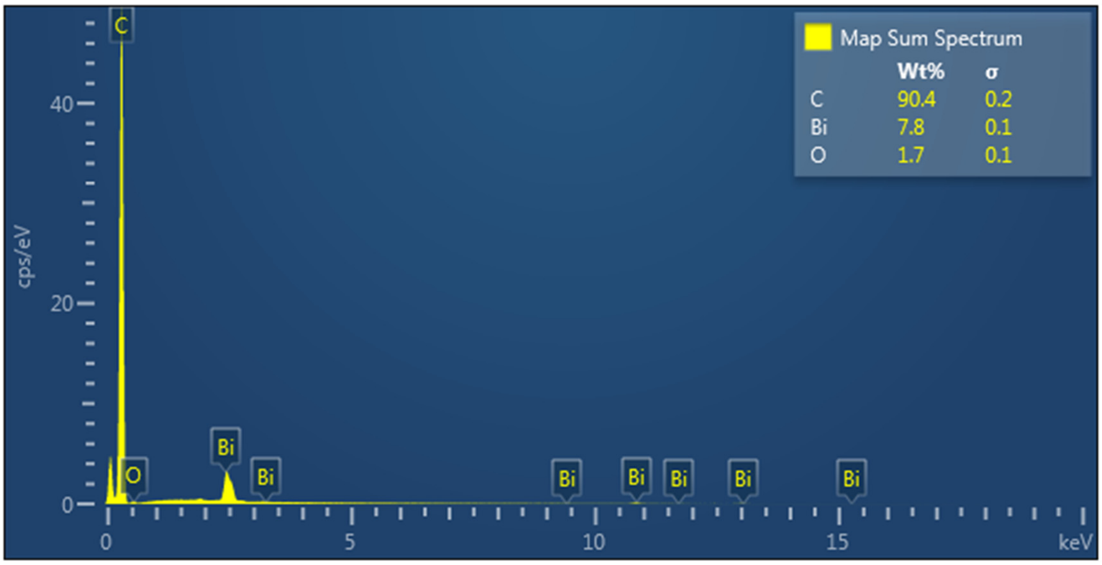

Element	Wt%	Wt% Sigma
C	90.42	0.15
O	1.74	0.11
Bi	7.84	0.12
Total:	100.00	

Figure S4. SEM-EDS images of LDPE composite containing a 10 wt% loading of Bi_2O_3.
